# The Changing Adoption of Culture Change Practices in U.S. Nursing Homes

**DOI:** 10.1093/geroni/igaa012

**Published:** 2020-06-05

**Authors:** Julie C Lima, Margot L Schwartz, Melissa A Clark, Susan C Miller

**Affiliations:** Department of Health Services, Policy & Practice, Brown University School of Public Health, Providence, Rhode Island

**Keywords:** Homelike environment, Pay for performance, Person-centered care, Resident-centered care, Staff empowerment

## Abstract

**Background and Objectives:**

The study aimed to: (i) describe whether culture change (CC) practice implementation related to physical environment, resident-centered care, and staff empowerment increased within the same nursing homes (NHs) over time; and (ii) identify factors associated with observed increases.

**Research Design and Methods:**

This was a nationally representative panel study of 1,584 U.S. NHs surveyed in 2009/2010 and 2016/2017. Survey data were merged with administrative, NH, and market-level data. Physical environment, staff empowerment, and resident-centered care domain scores were calculated at both time points. Multivariate logistic regression models examined factors associated with domain score increases.

**Results:**

Overall, 22% of NHs increased their physical environment scores over time, 32% their staff empowerment scores, and 44% their resident-centered care scores. However, 32%–68% of NHs with below median baseline scores improved their domain scores over time compared with only 11%–21% of NHs with baseline scores at or above the median. Overall, NHs in states with Medicaid pay-for-performance (with CC components), in community care retirement communities, with special care units and higher occupancy had significantly higher odds of increases in physical environment scores. Only baseline domain scores were associated with increases in staff empowerment and resident-centered care scores.

**Discussion and Implications:**

This is the first nationally representative panel study to assess NH CC adoption. Many NHs increased their CC practices, though numerous others did not. While financial incentives and indicators of financial resources were associated with increase in physical environment scores, factors associated with staff empowerment and resident-centered care improvements remain unclear. Studies are needed to assess whether the observed increases in CC adoption are associated with greater quality of life and care gains for residents and whether there is a threshold effect beyond which the efficacy of additional practice implementation may be less impactful.

**Translational Significance**

A growing number of studies show the benefits of culture change (CC) adoption in NHs, and federal regulations now emphasize the importance of resident-centered care. This study shows that many NHs continue to increase their CC activities, but increases are not universal. The lack of comprehensive increases likely speaks to the difficulty of change and the need for diverse facilitators to accomplish NH CC implementation for those NHs that are late adopters.

There are approximately 1.3 million nursing home (NH) residents on any given day in the United States (Harrington & Garfield, 2018). Improving the quality of life and care for these residents is an ongoing concern for researchers, policymakers, and providers. A nearly 20-year effort led by the two national NH associations (the American Health Care Association and Leading Age), the Pioneer Network (a national advocacy group founded in 1997), and Pioneer Network-affiliated state culture change (CC) coalitions has promoted what has become known as NH CC (Graham, 2012; Quality Partners of Rhode Island, 2005). Making NH care more patient- and family-centered is a hallmark of NH CC, and its intent is to achieve sustained quality improvement through practices that address fundamental change in the structure and functioning of NHs. As such, its primary focus is on the critical domains of Environmental Practices aimed at making NHs less institutional and more homelike (i.e., physical environment), Resident Care Practices to ensure care is more resident-centered/-directed (i.e., resident-centered care), and Workplace Practices intended to empower staff (i.e., staff empowerment; Graham, 2012).

Though more rigorous research support is still needed (Shier, Khodyakov, Cohen, Zimmerman, & Saliba, 2014; Zimmerman et al., 2016) and results of early analyses are mixed, there is evidence supporting the efficacy of NH CC implementation. A recent retrospective cohort study found Kansas NHs with greater participation in the state’s Medicaid CC pay-for-performance (P4P) program (PEAK 2.0; compared with nonparticipating NHs) had better performance on 7 of 13 quality indicators and on a composite measure (Hermer, Cornelison, Kaup, Poey, Stone, et al., 2018). This study also supported the presence of a dose–response association between practice implementation and resident depression; in NHs at higher stages of CC implementation (i.e., more practice implementation) the rates of depression were lower (compared with rates in nonparticipating NHs). Additionally, this and other panel studies have supported an association between NH CC implementation and reductions in Medicare/Medicaid survey deficiencies, improvement in many quality indicators, and higher resident satisfaction with quality of care and life (Grabowski, Elliot, Leitzell, Cohen, & Zimmerman, 2014; Miller, Lepore, Lima, Shield, & Tyler, 2014; Poey et al., 2017). On the other hand, a recent Veterans Administration (VA) NH panel study found no associations between greater CC implementation and improved quality (Sullivan, Shwartz, Stolzmann, Afable, & Burgess, 2018). As suggested by Sullivan and colleagues (2018), the lack of associations observed in the VA study may be due to the fact that implementation of CC practices was incentivized by the VA during the study period but performance on NH quality indicator outcomes was not. The same was true for the study conducted by Hermer, Cornelison, Kaup, Poey, Stone, and colleagues (2018), however. The variability in findings may also be related to differing rigor of the study designs, including the amount of time NHs were followed.

Despite the potential benefits of NH CC, there is substantial variation in the implementation of CC practices across the United States. In 2009/2010, we conducted a nationally representative survey of CC practice in more than 2,000 U.S. NHs. Eighty-five percent of responding NHs reported at least some CC practice adoption, though only 13% reported that CC had “completely changed the ways they care for residents” in all areas of the NH (Miller et al., 2018). In 2016/2017, a follow-up survey showed that these numbers had increased only slightly to 88% and 16%, respectively, but that 44% of NHs had complete adoption in CC across at least some portion of the NH, up from 33% in 2009/2010 (Miller et al., 2018).

In 2009/2010, we found that several characteristics were associated with higher CC scores. Compared with NHs in non-P4P states, NHs in states with Medicaid P4P that included CC quality measures had approximately twice the likelihood of having higher CC scores across the domains of physical environment, resident-centered care, and staff empowerment. NHs in states with Medicaid P4P, but without CC quality measures, had approximately 50% greater odds of having higher physical environment and staff empowerment scores. Similar to Grabowski and colleagues (2014), we found NHs in states with higher Medicaid reimbursement rates had higher levels of CC adoption, though only related to the physical environment domain (Miller et al., 2014). While no data were available to assess the association between Medicaid reimbursement rates and levels of CC adoption in our 2016/2017 follow-up study, we found that Medicaid P4P remained important (Miller et al., 2018). In addition to Medicaid payment policies, both a Kansas culture change study and a comparative study using a convenience sample of early CC adopters (compared with matched NH controls) found early adopters to be more often nonprofit and affiliated with Continuing Care Retirement Communities (CCRCs) as well as to have greater proportions of private-pay residents, higher nurse aide staffing rates, and higher occupancy rates (Grabowski et al., 2014; Hermer, Cornelison, Kaup, Poey, Stone, et al., 2018). However, a study of Kansas’s PEAK Medicaid P4P program found that over time the program appeared to enable expansion of CC adoption to NHs more representative of the states’ NHs overall (Hermer, Cornelison, Kaup, Poey, Drake, et al., 2018). Specifically, later NH adopters (compared with earlier adopters) were more likely to be for-profit, not affiliated with a CCRC, and to have lower occupancy rates (Hermer, Cornelison, Kaup, Poey, Drake, et al., 2018). Whether factors found to be associated with earlier NH adopters (but not necessarily later adopters) are associated with longitudinal increases in a NH’s CC practice adoption is of interest, but currently unknown.

In the current study, we compared baseline and follow-up data about CC practices from a nationally representative sample of 1,584 U.S. NHs surveyed in 2009/2010 and again in 2016/2017. The purpose of this research was twofold: (i) to determine rates of increase in CC implementation across the three CC domains of physical environment, resident-centered care and staff empowerment; and (ii) to identify the factors associated with the observed increases.

## Research Design and Methods

### Survey Data

The data for this project were from a national panel study of NHs surveyed in 2009/2010 (baseline) and again in 2016/2017 (when NHs with administrator 2009/2010 responses were re-surveyed). The 2009/2010 survey was part of a large Program Project designed to study broad topics related to NH quality. It was split into two survey instruments, one for NH administrators and the other for Directors of Nursing (DONs). The survey was sent to a stratified, proportionate random sample of 4,035 eligible U.S. NHs (i.e, Medicare and Medicaid certified NHs with at least 30 beds that had not taken part in any cognitive-based interviews during survey development); 2,215 NH administrators responded (response rate = 54.9%, cooperation rate = 62.6%).

The 2009/2010 survey included sets of items on the three critical CC domains of a NH’s physical environment, staff empowerment (both answered by NH administrators), and resident-centered care practices (answered by DONs). The number of items related to CC in this baseline survey was restricted because of the survey’s broad scope of collecting data for four unique studies within the Program Project, only one of which examined CC. Baseline survey items had strong internal consistency and validity (supported by agreement between survey responses and interviews; Miller et al., 2014).

The 2016/2017 follow-up “Nursing Home Culture Change Survey” was conducted under a separate grant focused entirely on CC. Surveys were sent to all NHs whose NH administrators responded at baseline. In this wave, NH administrators were chosen as the sole survey recipients given that our previous qualitative research showed administrators capable of answering CC practice questions (R. R. Shield, Looze, Tyler, Lepore, & Miller, 2014; Tyler, Shield, & Miller, 2015). Importantly, our analyses of cognitive interviews of DONs and NH administrators at the same NHs revealed administrators were better informed about CC-related practices and their nuances and therefore were the more credible respondents to report on these practices in their NHs (R. Shield, Tyler, Berridge, Clark, & Miller, 2018). Thus, the survey was sent to administrators at the 2,142 (of 2,215) NHs that were still operational. A response rate of 74% was obtained (n = 1,584). There was no detectable response bias for either survey wave, and so each wave is weighted solely for the sample design.

## Other Data Sources

### Variables of Interest

#### CC domain indices

While the 2016/2017 survey had a more comprehensive list of CC domains and items included within each domain, our change analyses focused on the three CC domains and items that could be comparably measured at both time periods—physical environment (six items), staff empowerment (seven items), and resident-centered care (four items). The physical environment index measured the extent to which NHs had a homelike atmosphere, including things like private rooms, play areas for children, common areas with personal items of residents on display, and unlimited access to the kitchen. The staff empowerment index measured the ability of staff to work together as well as opportunities for nursing assistants to be more involved in the care process. Items pertaining to the resident-centered care index focused on the extent to which residents had choices related to their activities and care.

The survey items and their point allocations for domain index scoring can be found in Supplementary Table 1. Additional detail can be found elsewhere (Miller et al., 2014; Tyler et al., 2011). Domain index scores were computed by summing the points for each question included in the domain. The physical environment score had a potential range of 0–14; staff empowerment 0–21; and resident-centered care 0–8. Each score was developed to represent a composite variable, or index, rather than a construct with underlying latent variables (Edwards & Bagozzi, 2000).

Each of the 34 items that made up the domain scores across both time points had some missing data, ranging from 1.8% to 9.9%. Items were considered missing at random as there is no theoretical reason to believe that missingness was related to CC improvement. In keeping with our earlier studies, missing responses were imputed within NHs using the NH’s domain-specific average based on the nonmissing items, rather than by other methods such as multiple imputation that would only use information on individual items across NHs and not consider other items within the domain index of a given NH. This imputation occurred only when a facility had two or fewer missing items for the physical environment (1.2% in 2009/2010, 1.4% in 2016/2017) and staff empowerment (3.0% and 5.8%, respectively) domains or one item for the resident-centered care domain (0.3% and 2.2%, respectively). We did not impute a missing score for one physical environment domain item whose response was not a Likert scale rating similar to the others in the domain (Supplementary Table 1). We imputed missingness similarly at the two time points, and thus any bias introduced by our imputation techniques is likely to be similar at both time points.

Among the 1,584 study NHs with surveys at both time points, there were 1,459 (92.1%) NHs with sufficient information to construct physical environment scores at both time points; data from 1,447 (91.4%) and 1,105 (69.8%) NHs were sufficiently complete for the staff empowerment and resident-centered scores, respectively. The resident-centered care domain had a lower starting sample size because these questions were asked of DONs rather than NH administrators at baseline, and only 76% of our eligible study NHs had DON surveys at baseline. Differences in characteristics between NHs that were dropped from analyses and those that were retained were minimal.

#### Outcome—Increase in CC over time

We were interested in assessing an increase in CC adoption over time. For both physical environment and staff empowerment, we identified an *increase* as being at least a three-point improvement in the score. This decision was based on what our investigators believed to be a meaningful increase during the time period between surveys. For the physical environment domain and staff empowerment domains, a three-point increase meant NHs had at least a one or two point increase on two or three survey items or had a three-point increase on one item. This ensured that NHs that moved from “no” to “working on it” or from “never” to “sometimes” on just a single item or two were not considered improved because they had several years between surveys to make improvements. Due to the smaller number of items and range of scores, an increase of two or more points was considered an increase for the resident-centered care domain. Therefore, NHs had to have moved from “no” to “yes” on one of the four items or from “no” to “working on it” on at least two items to be considered improved. A review of the indices’ distributions supported our decision.

#### Structure and staffing characteristics

Of interest were the factors associated with a NH’s increase in CC practice over time. We focused primarily on baseline values because increases in CC adoption over time may have led to subsequent changes to these same variables (i.e., staffing, case mix, other). Models included several variables that have previously been associated with CC adoption as well as variables reflective of a NH’s case mix (derived from resident-level data). Using the baseline survey, we included a measure of NH administrator tenure categorized as 0–2 years, more than 2–7 years, and more than 7 years. Whether the NH was part of a CCRC at baseline came from the 2010 NH Compare (https://www.medicare.gov/nursinghomecompare/search.html). Additional structure and staffing variables were taken or derived from 2009 and 2010 Online Survey Certification and Reporting (OSCAR) data. These variables included profit status; the presence of any special care unit (excluding a ventilator unit); registered nurse, licensed practical nurse, and certified nursing assistant hours per resident day (all standardized); number of NH beds (80 or fewer vs more than 80); occupancy rate; and the proportion of residents with Medicare and Medicaid as payers.

#### State policy and regional characteristics

State policy and regional characteristics were also obtained from several sources. The presence of Medicaid P4P programs (with and without a CC focus) was captured at baseline and subsequently. We obtained baseline information on P4P from a 2011 State Policy survey conducted in conjunction with the original Program grant (Miller et al., 2014). Information post 2011 was retrieved from a report by the Medicaid and CHIP Payment and Access Commission (MACPAC) and supplemented by Medicaid website reviews and conversations with state policy experts (MACPAC, 2014; Miller et al., 2018). The resulting variable was dichotomous: states having Medicaid P4P with CC quality measures at one or more time points (Kansas, Ohio, Colorado, Oklahoma, and Utah) compared with states without such programs (all others).

Staff at the Pioneer Network reviewed the state CC coalitions’ 2015 activity reports (of coalition-sponsored educational and mentoring events), and for states with coalitions, reported to study investigators a coalition’s level of activity in 2015 (C. Lieblich, personal communication, February 2018). Based on these reports, we created a flag of states with very active coalitions (Arkansas, California, Georgia, Illinois, Louisiana, Missouri, North Carolina, New Jersey, Ohio, Oregon, Pennsylvania, Rhode Island). The average 2009 Medicaid NH reimbursement rate was taken from the 2011 State policy survey. More current rate information was unavailable. A standardized (baseline) Herfindahl–Hirschman Index to quantify county-level NH competition was derived from OSCAR data. A low score on this index indicates more NH competition in the county. Finally, an indicator of urban/rural status was taken from the Area Resource File.

#### Aggregated resident characteristics

To control for resident case mix, baseline resident characteristics were modeled after prevalence measures found in Long-Term Care Facts on Care in the United States data set (Brown University School of Public Health, 2019). Using a residential history file methodology (Intrator, Hiris, Berg, Miller, & Mor, 2011), we captured a prevalent cohort of all persons present in the NHs on the first Thursday in April of the baseline survey year. We then used their closest NH assessments and Medicare enrollment files from the Centers for Medicare and Medicaid Services (CMS) to create several facility-aggregated resident characteristics. These characteristics included percent of residents who were black, average age, average standardized Resource Utilization Group Nursing Case Mix Index, average activities of daily living score (range of 0–28), and the percent of residents with severe cognitive impairment (i.e., five or six on the Cognitive Performance Scale).

### Statistical Analyses

Survey procedures in Stata 15 (StataCorp, 2017) were used throughout to adjust for the sampling design, though the sample sizes reported reflect unweighted numbers. The sampling design consisted of 19 strata that reflected: (i) the size of the NH population in a state (smaller or larger, determined by the number of freestanding NHs in the state); (ii) owner type (for-profit, not-for-profit, or hospital based); (iii) bed size (small 30–120 beds, or large 120+ beds); and (iv) percentage of nonwhite residents (≤10% vs >10%). As appropriate, 2009/2010 probability weights were used to report baseline characteristics and 2016/2017 probability weights were used in our multivariate analyses. Because there was no detection of nonresponse bias in either wave of the study, the weight at each time point simply reflected the stratum-specific, nonresponse adjusted weight. More complete information regarding survey design at each wave can be found elsewhere (Miller et al., 2014, 2018). Multivariate analyses were clustered on state. Logistic regression was used to examine factors associated with increase (vs no increase) in the domain scores.

The study was reviewed and approved by Brown University’s Institutional Review Board (#1703001723). The use of CMS data was covered under the strict terms of a Data Use Agreement (DUA) and included the appropriate Waiver of Informed Consent and HIPAA Waiver of Authorization for the use of person-level claims, enrollment, and assessment data. In compliance with our funder’s Resource Sharing Policy, while person level data cannot be shared due to DUA restrictions, additional information about the data and methods used for these analyses can be found in Brown’s Digital Repository (https://repository.library.brown.edu/studio/item/bdr:847097/).

## Results

### NH Characteristics and CC Scores

**Figure 1.**
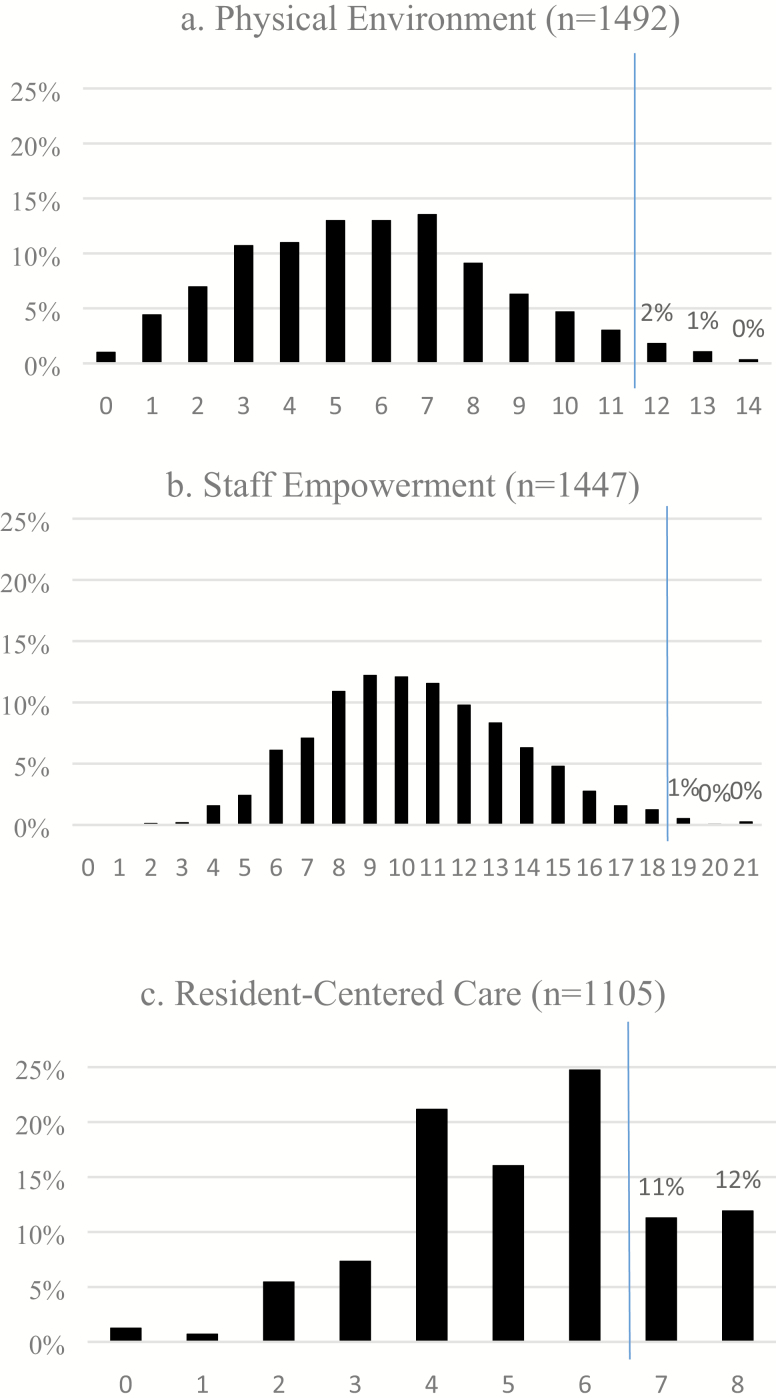
Baseline domain scores. *Note.* Bars to the right of the vertical line show facilities whose baseline scores were so high that they were removed from subsequent analyses.

Table 1 provides weighted baseline characteristics for the overall sample (i.e., those with NH administrator surveys at both time points). Figure 1 shows the distribution of baseline responses for NHs with domain-specific scores available at both time points. As indicated by the vertical lines in the graph, 3% and 1% of NHs scored high enough at baseline on the physical environment and staff empowerment scores, respectively, to be removed from further analyses because there was no room to increase CC adoption further in those NHs according to our definition. In contrast, 23% of NHs scored high enough at baseline on the resident-centered score to be removed from further analyses. Facilities remaining in the resident-centered care analyses were less likely than the full sample to be for-profit (66% vs 69%, *p* < .05—not shown) but did not differ from the full sample on any other measured characteristics.

**Table 1.** Characteristics of Overall Study Sample

Characteristics	Full sample (*n* = 1,564) % or Mean (*SE*)
Facility characteristics	
Administrator tenure	
0–2 years	40.1
>2–7 years	33.9
>7 years	26.1
Continuous care retirement community	9.5
For profit	69.1
Small facility (80 or fewer beds)	34.4
Occupancy rate	85.0 (0.33)
Any special unit	20.8
RN hours per resident day	0.4 (0.01)
LPN hours per resident day	0.8 (0.01)
CNA hours per resident day	2.3 (0.02)
Percent with Medicare	13.9
Percent with Medicaid	61.0
*State policy and regional characteristics*	
State pay for performance	
No P4P or P4P without CC at either time	87.9
Any P4P with CC	12.1
State culture change coalition (very active)	37.5
Medicaid rate 2009	$160.28 (0.76)
County Herfindahl–Hirschman Index	0.2 (0.01)
Nursing home in urban county	66.3
*Resident case mix*	
Percent black residents	
None	38.5
Below median	8.3
Above median	53.3
Average age	81.0 (0.16)
Average RUGS Case Mix Index	0.8 (0.02)
Average ADL	16.4 (0.07)
Percent high CPS	17.1

*Note*. ADL = activities of daily living; CC = culture change; CNA = certified nursing assistant; CPS = cognitive performance scale; LPN = licensed practical nurse; P4P = Medicaid pay for performance; RN = registered nurse; RUGS = Resource Utilization Group; *SE* = standard error.

**Figure 2.**
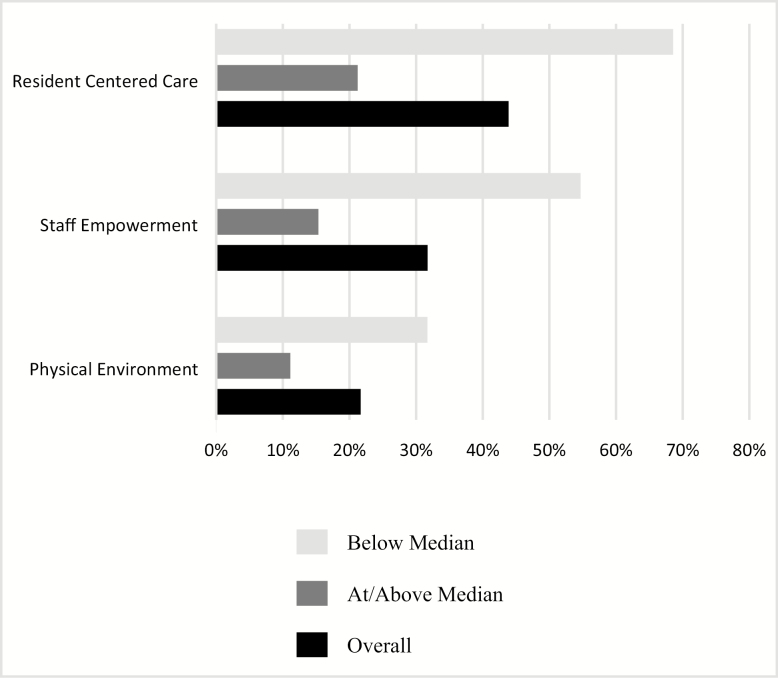
Percent of nursing homes with increased domain scores over time, overall and stratified by baseline scores.

Figure 2 displays increases in CC scores over time among NHs with room to increase. Overall, 22% of NHs increased their physical environment scores over time, 32% their staff empowerment scores, and 44% their resident-centered care scores. When NHs were stratified by baseline score, nearly one-third (32%) of NHs that scored below the median at baseline in the physical environment domain increased their score, 55% of NHs did so within the staff empowerment domain, and 68% within the resident-centered care domain. Among NHs that scored at or above the median at baseline, 11%–21% of NHs showed at least some increase at follow-up. Supplementary Tables 2–4 present baseline characteristics of facilities that did and did not increase their domain score over time, stratified by baseline domain scores.

### Predictors of Increased CC Adoption Over Time—Multivariate Analyses

Overall, the strongest association with an increase in CC score over time within any model in Table 2 was a NH’s baseline score on that domain. NHs with higher domain scores at baseline were significantly less likely than those with lower domain scores to increase their scores further by Time 2 (all associations significant at *p* < .001). No additional associations were found for the staff empowerment and resident-centered care improvement scores. There were several factors, however, that were associated with an improvement in the physical empowerment domain over time.

**Table 2.** Logistic Regression of Nursing Home and Market Characteristics on Culture Change Improvement Across Three Domains

Variable	Physical environment	Staff empowerment	Resident-centered care
	Any vs none	Any vs none	Any vs none
	(*n* = 1,402)	(*n* = 1,427)	(*n* = 842)
	OR (95% CI)	OR (95% CI)	OR (95% CI)
Baseline domain score	0.65***	0.64***	0.37***
	(0.60, 0.70)	(0.61, 0.67)	(0.32, 0.43)
Administrator tenure			
0–2 years	Ref	Ref	Ref
>2–7 years	1.15	1.11	1.05
	(0.82, 1.61)	(0.79, 1.55)	(0.68, 1.61)
>7 years	0.70	0.86	1.48
	(0.45, 1.10)	(0.60, 1.22)	(0.92, 2.39)
Continuous care retirement community	1.87*	0.72	0.82
	(1.07, 3.27)	(0.42, 1.22)	(0.46, 1.47)
For profit	0.88	1.15	1.07
	(0.60, 1.27)	(0.84, 1.57)	(0.69, 1.64)
80 or fewer beds in NH	1.12	1.14	0.90
	(0.76, 1.66)	(0.79, 1.65)	(0.63, 1.29)
Occupancy rate (5 point increase)	1.07*	1.03	1.01
	(1.00, 1.14)	(0.97, 1.09)	(0.94, 1.08)
Any special care unit	1.51*	1.13	1.09
	(1.02, 2.23)	(0.80, 1.59)	(0.73, 1.62)
RN hours per resident day	1.10	1.07	1.07
	(0.92, 1.30)	(0.89, 1.27)	(0.88, 1.32)
LPN hours per resident day	1.14	0.98	0.97
	(0.97, 1.34)	(0.84, 1.16)	(0.80, 1.18)
CNA hours per resident day	0.97	0.97	1.14
	(0.82, 1.16)	(0.84, 1.12)	(0.94, 1.39)
State pay for performance with CC	1.86**	1.39	1.28
	(1.25, 2.77)	(0.93, 2.07)	(0.76, 2.15)
State culture change coalition	0.75	0.89	1.25
	(0.54, 1.03)	(0.64, 1.23)	(0.88, 1.76)
Average Medicaid nursing home reimbursement rate	1.01	1.00	0.95
	(0.96, 1.07)	(0.95, 1.05)	(0.89, 1.01)
County Hirschman–Herfindahl Index	1.14	0.99	1.01
	(0.97, 1.34)	(0.83, 1.19)	(0.81, 1.25)
Urban (vs rural) area	1.00	1.03	1.34
	(0.67, 1.49)	(0.72, 1.48)	(0.89, 2.01)
Percent black residents			
Lowest decile	Ref	Ref	Ref
Below median	0.63	0.80	0.93
	(0.35, 1.12)	(0.48, 1.33)	(0.49, 1.78)
Above median	0.70	1.07	0.76
	(0.48, 1.01)	(0.77, 1.51)	(0.48, 1.20)
Percent with Medicare	1.00	0.99	1.01
	(0.99, 1.02)	(0.98, 1.00)	(0.99, 1.02)
Percent with Medicaid	1.00	1.00	1.00
	(0.99, 1.01)	(0.99, 1.01)	(0.99, 1.02)
Average age	1.02	1.00	1.00
	(0.98, 1.05)	(0.97, 1.02)	(0.96, 1.04)
Average RUGS Case Mix	0.87	1.00	0.84
	(0.69, 1.10)	(0.84, 1.18)	(0.65, 1.09)
Average number of ADL impairments	0.96	1.02	1.05
	(0.89, 1.04)	(0.96, 1.09)	(0.96, 1.14)
Percent of resident with high CPS	1.00	0.99	1.00
	(0.99, 1.01)	(0.98, 1.00)	(0.98, 1.01)

*Note*. ADL = activities of daily living; CC = culture change; CI = confidence interval; CNA = certified nursing assistant; CPS = cognitive performance scale; NH = nursing home; LPN = licensed practical nurse; OR = odds ratio; Ref = reference; RN = registered nurse; RUGS = Resource Utilization Group; .

*p < .05. **p < .01. ***p < .001.

Controlling for baseline domain score and other factors, being part of a CCRC, increased occupancy rate, having a special care unit, and residing in a state with a Medicaid P4P program with a CC component were each associated with an improvement in the physical environment domain score. Specifically, the odds were nearly twice as likely in NHs that were part of a CCRC compared with those that were not (1.87, *p* < .05) to have increased physical environment domain scores over time. A five-point increase in occupancy rate was associated with a significant 1.07 adjusted odds ratio (*p* < .05) of improvement. Facilities with a special care unit (excluding ventilators) had 1.51 times the odds (*p* < .05) as those without such a unit of showing improvements in their physical environment score. Finally, NHs in a state with Medicaid P4P programs with CC quality measures had 1.86 times the odds of having an increased physical environment score (*p* < .01) compared with NHs in states without such P4P programs.

## Discussion and Implications

This was the first nationally representative panel study to assess adoption of CC practices within NHs over time. The proportionate random sample design combined with rigorous survey collection methods leading to higher response rates than many other national NH surveys resulted in a final sample that essentially mirrors NHs nationally. For example, the proportion of NHs by profit status and their staffing hours and proportion of stays paid for by Medicaid are nearly identical to those reported by the National Center for Health Statistics (Harris-Kojetin et al., 2019). This is a major improvement over most studies of CC.

We found that many NHs increased their adoption of CC practices between 2009/2010 and 2016/2017 within the three domains examined. However, increases were far from universal and there remains ample room for continued improvement. Study findings both illuminate the difficulty of widespread NH change as well as highlight the need for diverse efforts (incentives and others) to accomplish widespread NH CC.

There were no consistent factors associated with increases in CC implementation across domains aside from baseline score. We believe the observed negative associations between a higher baseline domain score and odds of improvement reflects that NHs with higher baseline scores have less opportunity for improvement overall (Figure 2). Although we removed NHs with no opportunity to improve, the remaining NHs that scored at or above the median at baseline showed less improvement than those that scored below the median.

No other measured facility, state policy, regional, or facility-level resident case mix characteristics were statistically significantly associated with improvements in staff empowerment or resident-centered care CC practices over time. There were several factors associated with improvements in physical environment over, however. Controlling for baseline domain score and other factors, being part of a CCRC, increased occupancy rate, having a special care unit, and residing in a state with a Medicaid P4P program with a CC component were each associated with an improvement in the physical environment domain score. In general, these characteristics serve as proxies for higher facility resources/quality compared with NHs without these characteristics (e.g., Joyce, McGuire, Bartels, Mitchell, & Grabowski, 2018).

More complete CC adoption in the physical environment domain most certainly requires additional capital to make the necessary adjustments to the physical layout of the NH, necessary staffing updates to make the kitchen more accessible and dining times more flexible, and time for the changes to be implemented (Hermer, Cornelison, Kaup, Poey, Stone, et al., 2018). The physical environment domain may be the most obvious area in which to show sustained improvements within a P4P incentive program. Practices such as having single rooms or an open dining plan are concrete and once incentivized may more easily survive staff and leadership turnover. Conversely, practices in the staff empowerment and resident-centered care domains require a continual buy-in on the part of leadership and staff, families, and residents. Therefore, although previous studies have found Medicaid P4P with CC quality measures are significantly associated with adoption of resident-centered care and staff empowerment practices (Miller et al., 2014; Hermer, Cornelison, Kaup, Poey, Drake, et al., 2018), other factors such as leadership style and/or turnover may make changes unsustainable and further change unlikely (Bowers, Nolet, & Jacobson, 2016). Our data show some support for this suggestion. Among the 12% of facilities in our overall sample that we determined to have P4P with CC, only 25% had it at both baseline and Time 2, while 75% implemented it sometime postbaseline. Looking at these two groups separately, those with P4P with CC at baseline did have higher baseline staff empowerment scores than facilities that implemented policy later, but on average they were not able to sustain these baseline levels over time. Though still not reaching statistical significance in the multivariate model, it appears that those that implemented P4P with CC more recently had larger increases between baseline and Time 2 of staff empowerment than those that had received P4P with CC incentives earlier. This suggests continuing improvement may be more difficult than initial improvement, perhaps because initial improvement addresses practices more easily attainable and because innovative leadership or staff stability may be difficult to sustain. Similarly, those that received P4P more recently made larger gains in the resident-centered care domain between baseline and Time 2 (results not shown).

Still, even given the above, it is important to note that the null findings regarding the association between increases in CC scores and being in a state having Medicaid P4P with CC incentives may in part reflect a lack of power to detect significant differences. For staff empowerment practices, in particular, Supplementary Table 3 shows a higher proportion of NHs with increases in scores (compared with NHs without increased scores) resided in states with Medicaid P4P with CC incentives. In fact, descriptively, the magnitude of difference observed in Supplementary Table 3 is similar to that observed in Supplementary Table 2 that depicts descriptive differences for NH’s with and without increases in the physical environment CC scores.

Sturdevant, Mueller, and Buckwalter (2018) suggest the importance of additional training for providers and direct care workers in the implementation of CC practices in general, as well as continued input from early adopters at association meetings and the like to encourage those daunted by the idea of making a change. This may be particularly relevant to the staff empowerment domain. While part of its intention is to provide greater incentives to employees for additional training, the staff empowerment domain also requires a vested buy-in of the CC paradigm shift by all staff and administrators (Sturdevant et al., 2018). It requires staff to take on new responsibilities (e.g., cross-training in another area) and potentially learn a new way of interacting with coworkers and the residents under their care. Therefore, for NHs that are not yet fully invested, it may be difficult to maintain momentum toward higher levels of staff empowerment.

Only 11% of NHs that scored at or above the median at baseline (Figure 2) showed improvement in their physical environment scores, and these scores left ample room for additional improvement (mean domain score at Time 2 was 8, with a possible maximum of 14, results not shown). It remains to be seen whether improvements in the physical environment beyond a certain point are necessary, however. A recent study suggests that physical environment modifications such as the overall dining experience and the provision of delicious food whenever desired may be of more benefit to residents’ psychosocial well-being than changes to the actual physical structure, such as those needed to create small households (Hermer, Bryant, Pucciarello, Mlynarczyk, & Zhong, 2017). If this is the case, then NHs with lower financial means can be less concerned about making the more expensive changes but instead should focus on making the most of the changes they are financially able to make (Hermer et al., 2017).

This study has limitations that deserve comment. First, it focused on only three of the six CC domains suggested by the HATCh model (Quality Partners of Rhode Island, 2005), and within each we included only a limited number of items that were available at both time points. Resident-centered care, in particular, had only four items and scores were already high at baseline. Almost a quarter of NHs were excluded from the resident-centered care change analyses because they scored so high (7 or 8) at baseline that they were unable to improve further by our definition. Among all eligible NHs with scores at both time points, 18% (not shown) scored the maximum score by follow-up. However, the indices used had good measurement properties, including construct validity (Bott et al., 2009; Doty, Koren, & Sturla, 2008; Miller et al., 2014; Mueller, 2007). The 2016/2017 survey included additional domains and expanded the number of items included for each of the three domains studied here. Thus, other ongoing research concentrating on the 2016/2017 survey only will be able to assess CC practice adoption more comprehensively cross-sectionally. Second, responses may reflect some social desirability bias on the part of NH administrators. While we cannot discount this possibility, our past work has shown that NH administrators may show less bias in their responses to our questions than DONs (R. Shield et al., 2018). Third, because of the shift in study design between baseline and follow-up, resident-centered care responses were provided by DONs at baseline, and by NH administrators at follow-up. This resulted in a reduced sample size for analyses and may have introduced some measurement error. If DONs are subject to more social desirability bias, then baseline scores were potentially inflated compared with follow-up scores.

This study provides the first longitudinal assessment of CC adoption at a national level. Many NHs are continuing to increase their CC activities, though there remains ample room for continued growth. Further studies are needed to assess whether these increases in adoption are associated with greater quality of life and care gains for residents, as well as whether there is a threshold effect beyond which the efficacy of additional practice implementation may be less impactful.

## Funding

This work was supported by the National Institutes of Health, National Institute on Aging [R01AG048940].

## Supplementary Material

igaa012_suppl_Supplementary_Material
