# Supplementary material for: The Changing Adoption of Culture Change Practices in U.S. Nursing Homes
Source: Innov Aging. 2020 Jun 5;4(3):igaa012. doi: 10.1093/geroni/igaa012 (PMC7272786; doi:10.1093/geroni/igaa012)
Supplement: igaa012_suppl_Supplementary_Material [file igaa012_suppl_supplementary_material.docx]

**Supplementary Material**

**Supplement Table 1. Domain Score Coding and Sample Sizes for NHs with Administrator Surveys at Both Baseline and Follow-up (n=1,564)**

| Item | Scoring |
| --- | --- |
| Physical Environment Domain (n=1,459) | |
| What proportion of residents have a private room? | 0=0%; 1=1 to 4%; 2=5 to 25%; 3=26 to 75%; 4=76 to 100% |
| *Please indicate if each of the following statements applies to your facility:* |  |
| The lighting, furniture, and overall environment in residents' living areas are similar to what we would use in our own homes | 0= no; 1= working on it; 2= yes |
| We have kitchen areas that are accessible to residents and families 24/7 | 0= no; 1= working on it; 2= yes |
| We display residents' personal items, such as family photos, in common living areas outside of their rooms | 0= no; 1= working on it; 2= yes |
| We have indoor and/or outdoor play areas for children | 0= no; 1= working on it; 2= yes |
| We use open dining where a meal is available for AT LEAST A TWO HOUR period during which residents can choose when to eat | 0= no; 1= working on it; 2= yes |
| Staff Empowerment (n=1,447) | |
| *In your facility, how often…* |  |
| Does staff work together to cover shifts when someone can't come to work? | 0=never; 1=sometimes 2=often; 3=always |
| Is staff cross-trained to perform tasks outside of their assigned job duties, such as housekeeping staff trained to provide feeding assistance or nursing assistants trained to provide activities? | 0=never; 1=sometimes 2=often; 3=always |
| Is staff, other than activity and management staff, involved in planning social events? | 0=never; 1=sometimes 2=often; 3=always |
| Do nursing assistants take part in quality improvement teams? | 0=never; 1=sometimes 2=often; 3=always |
| Do nursing assistants know when a resident’s care plan has changed? | 0=never; 1=sometimes 2=often; 3=always |
| Does your nursing home give bonuses, raises, or other rewards to nursing assistants who receive extra training or education? | 0=never; 1=sometimes 2=often; 3=always |
| Does your nursing home permit nursing assistants to choose which residents they care for? | 0=never; 1=sometimes 2=often; 3=always |
| Resident-centered Care (n=1,105) | |
| *At the present time, is it the practice in your facility that…* |  |
| Residents choose the times they prefer to eat? | 0= no; 1= working on it; 2= yes |
| Residents choose when they want to get up in the morning? | 0= no; 1= working on it; 2= yes |
| Residents participate in choosing the types of activities that are offered to them? | 0= no; 1= working on it; 2= yes |
| Residents participate in deciding which nursing assistants are assigned to care for them? | 0= no; 1= working on it; 2= yes |

**Supplemental Table 2.1 Baseline Characteristics of Facilities that Did and Did Not Increase in the Physical Environment Domain Over Time, Stratified by Baseline Domain Score**

|  | **Increased Over Time** | | **Did Not Increase Over Time** | |
| --- | --- | --- | --- | --- |
|  | **Score BM** | **A/A M** | **BM** | **A/A M** |
|  | **n=224** | **n=85** | **n=465** | **n=642** |
|  | **%/mean (s.e.)** | **%/mean (s.e.)** | **%/mean (s.e.)** | **%/mean (s.e.)** |
| Administrator tenure |  |  |  |  |
| 0 to 2 years | 45.2% | 30.3% | 44.4% | 36.1% |
| More than 2 to 7 years | 38.0% | 39.2% | 32.0% | 34.4% |
| More than 7 years | 16.8% | 30.6% | 23.6% | 29.5% |
| Continuous care retirement community | 5.8% | 20.0% | 4.1% | 10.3% |
| For profit | 76.9% | 52.1% | 80.4% | 64.4% |
| Small Facility (80 or fewer beds) | 34.0% | 43.5% | 26.3% | 36.9% |
| Occupancy rate | 85.57 (0.80) | 86.70 (1.27) | 84.25 (0.62) | 84.81 (0.55) |
| Any special unit | 20.1% | 27.0% | 15.6% | 23.3% |
| RN Hours per resident day | 0.33 (0.01) | 0.40 (0.03) | 0.33 (0.01) | 0.38 (0.01) |
| LPN Hours per resident day | 0.82 (0.02) | 0.86 (0.04) | 0.83 (0.01) | 0.79 (0.01) |
| CNA Hours per resident day | 2.27 (0.04) | 2.43 (0.07) | 2.30 (0.03) | 2.33 (0.03) |
| Percent with Medicare | 13.04 (0.60) | 16.58 (1.89) | 13.96 (0.48) | 13.98 (0.52) |
| Percent with Medicaid | 66.16 (1.08) | 50.00 (2.59) | 65.90 (0.83) | 58.21 (0.80) |
| ***State Policy and Regional Characteristics*** |  |  |  |  |
| State Pay for Performance |  |  |  |  |
| No P4P or P4P without CC at either time | 88.0% | 80.2% | 91.6% | 87.1% |
| Any P4P with CC | 12.0% | 19.8% | 8.4% | 12.9% |
| State culture change coalition (very active) | 35.6% | 37.0% | 44.5% | 36.3% |
| Medicaid Rate 2009 | $160.00 (1.99) | $160.03 (3.13) | $158.68 (1.37) | $160.55 (1.25) |
| County Herfindahl-Hirschman Index | 0.23 (0.19) | 0.28 (0.03) | 0.20 (0.01) | 0.22 (0.01) |
| Nursing home in urban county | 66.9% | 62.7% | 73.7% | 63.5% |
| ***Resident Case Mix*** |  |  |  |  |
| Percent Black residents |  |  |  |  |
| None | 32.1% | 60.6% | 24.7% | 44.8% |
| Below median | 7.5% | 3.5% | 7.3% | 9.8% |
| Above median | 60.5% | 35.8% | 68.0% | 45.4% |
| Average age | 79.67 (0.42) | 83.86 (0.49) | 79.38 (0.30) | 81.89 (0.25) |
| Average RUGS Case Mix Index | 0.83 (0.01) | 0.82 (0.01) | 0.85 (0.00) | 0.83 (0.00) |
| Average ADL | 16.27 (0.20) | 16.46 (0.30) | 16.69 (0.14) | 16.34 (0.11) |
| Percent high CPS | 16.61 (0.85) | 17.31 (1.10) | 17.58 (0.56) | 17.11 (0.50) |

*Notes*. BM=below median; A/A M= at/above median; RN=registered nurse; LPN=licensed practical nurse; CNA=certified nursing assistant; P4P=Medicaid pay for performance; CC=culture change; RUGS=Resource Utilization Group; ADL=activities of daily living; CPS=cognitive performance scale

**Supplemental Table 2.2 Baseline Characteristics of Facilities that Did and Did Not Increase in the Staff Empowerment Domain Over Time, Stratified by Baseline Domain Score**

|  | **Increased Over Time** | | **Did Not Increase Over Time** | |
| --- | --- | --- | --- | --- |
|  | **BM** | **A/A M** | **BM** | **A/A M** |
|  | **n=315** | **n=131** | **n=272** | **n=719** |
|  | **%/mean (s.e.)** | **%/mean (s.e.)** | **%/mean (s.e.)** | **%/mean (s.e.)** |
| Administrator tenure |  |  |  |  |
| 0 to 2 years | 47.5% | 36.3% | 44.0% | 35.4% |
| More than 2 to 7 years | 34.0% | 36.7% | 30.4% | 35.5% |
| More than 7 years | 18.5% | 27.0% | 25.7% | 29.1% |
| Continuous care retirement community | 8.7% | 5.5% | 11.5% | 8.5% |
| For profit | 70.8% | 74.7% | 67.6% | 69.6% |
| Small Facility (80 or fewer beds) | 31.2% | 39.6% | 35.8% | 33.3% |
| Occupancy rate | 84.77 (0.74) | 85.79 (1.11) | 85.22 (0.79) | 84.85 (0.51) |
| Any special unit | 21.8% | 14.3% | 18.2% | 21.7% |
| RN Hours per resident day | 0.37 (0.02) | 0.36 (0.02) | 0.35 (0.01) | 0.36 (0.01) |
| LPN Hours per resident day | 0.81 (0.02) | 0.79 (0.02) | 0.81 (0.02) | 0.81 (0.01) |
| CNA Hours per resident day | 2.29 (0.03) | 2.31 (0.05) | 2.31 (0.03) | 2.34 (0.02) |
| Percent with Medicare | 13.75 (0.66) | 13.71 (0.95) | 14.22 (0.79) | 13.89 (0.43) |
| Percent with Medicaid | 63.04 (1.09) | 60.82 (1.79) | 61.82 (1.28) | 60.67 (0.71) |
| ***State Policy and Regional Characteristics*** |  |  |  |  |
| State pay for performance |  |  |  |  |
| No P4P or P4P without CC at either time | 87.4% | 82.8% | 92.3% | 87.1% |
| Any P4P with CC | 12.6% | 17.2% | 7.7% | 12.9% |
| State culture change coalition (very active) | 37.7% | 33.5% | 37.8% | 38.6% |
| Medicaid Rate 2009 | $161.63 (1.72) | $159.83 (2.89) | $161.66 (2.03) | $158.78 (1.06) |
| County Herfindahl-Hirschman Index | 0.20 (0.01) | 0.23 (0.02) | 0.21 (0.01) | 0.24 (0.01) |
| Nursing home in urban county | 72.8% | 60.1% | 68.8% | 64.3% |
| ***Resident Case Mix*** |  |  |  |  |
| Percent Black residents |  |  |  |  |
| None | 30.8% | 42.5% | 34.2% | 40.8% |
| Below median | 7.3% | 6.6% | 8.3% | 8.4% |
| Above median | 62.0% | 50.9% | 57.5% | 50.8% |
| Average age | 79.67 (0.41) | 81.62 (0.55) | 81.01 (0.41) | 81.24 (0.21) |
| Average RUGS Case Mix Index | 0.84 (0.01) | 0.83 (0.01) | 0.83 (0.01) | 0.83 (0.00) |
| Average ADL | 16.31 (0.18) | 16.62 (0.26) | 16.49 (0.19) | 16.40 (0.11) |
| Percent high CPS | 15.56 (0.62) | 16.97 (1.06) | 17.36 (0.71) | 18.08 (0.49) |

*Notes*. BM=below median; A/A M= at/above median; RN=registered nurse; LPN=licensed practical nurse; CNA=certified nursing assistant; P4P=Medicaid pay for performance; CC=culture change; RUGS=Resource Utilization Group; ADL=activities of daily living; CPS=cognitive performance scale

**Supplemental Table 2.3 Baseline Characteristics of Facilities that Did and Did Not Increase in the Resident Centered Care Domain Over Time, Stratified by Baseline Domain Score**

|  | **Increased Over Time** | | **Did Not Increase Over Time** | |
| --- | --- | --- | --- | --- |
|  | **BM** | **A/A M** | **BM** | **A/A M** |
|  | **n=275** | **n=95** | **n=123** | **n=356** |
|  | **%/mean (s.e.)** | **%/mean (s.e.)** | **%/mean (s.e.)** | **%/mean (s.e.)** |
| Administrator Tenure |  |  |  |  |
| 0 to 2 years | 39.2% | 33.5% | 43.8% | 36.7% |
| More than 2 to 7 years | 37.1% | 29.1% | 35.6% | 39.4% |
| More than 7 years | 23.7% | 37.4% | 20.6% | 23.9% |
| Continuous care retirement community | 8.1% | 11.3% | 10.4% | 12.0% |
| For profit | 68.3% | 66.3% | 70.5% | 64.9% |
| Small facility (80 or fewer beds) | 35.2% | 34.9% | 38.0% | 36.5% |
| Occupancy rate | 84.13 (0.81) | 85.76 (1.27) | 85.94 (1.15) | 84.76 (0.72) |
| Any special unit | 20.8% | 18.9% | 13.9% | 24.4% |
| RN Hours per resident day | 0.36 (0.02) | 0.26 (0.02) | 0.35 (0.02) | 0.36 (0.01) |
| LPN Hours per resident day | 0.82 (0.02) | 0.79 (0.02) | 0.80 (0.02) | 0.80 (0.02) |
| CNA Hours per resident day | 2.33 (0.03) | 2.39 (0.06) | 2.21 (0.05) | 2.34 (0.04) |
| Percent with Medicare | 13.17 (0.67) | 13.50 (0.93) | 13.06 (0.92) | 13.63 (0.62) |
| Percent with Medicaid | 63.19 (1.11) | 61.45 (1.80) | 62.21 (1.89) | 60.27 (0.99) |
| ***State Policy and Regional Characteristics*** |  |  |  |  |
| State pay for performance |  |  |  |  |
| No P4P or P4P without CC at either time | 87.6% | 86.4% | 86.6% | 88.6% |
| Any P4P with CC | 12.4% | 13.7% | 13.4% | 11.4% |
| State culture change coalition (very active) | 43.7% | 31.1% | 33.1% | 32.0% |
| Medicaid Rate 2009 | $157.85 (1.67) | $159.21 (2.77) | $162.45 (2.97) | $160.15 (1.67) |
| County Herfindahl-Hirschman Index | 0.23 (0.02) | 0.22 (0.02) | 0.22 (0.02) | 0.23 (0.01) |
| Nursing home in urban county | 65.3% | 66.4% | 63.5% | 61.0% |
| ***Resident Case Mix*** |  |  |  |  |
| Percent Black residents |  |  |  |  |
| None | 38.2% | 40.9% | 31.4% | 45.3% |
| Below median | 6.4% | 10.9% | 5.9% | 10.1% |
| Above median | 55.5% | 48.2% | 62.7% | 44.6% |
| Average age | 80.6 (0.40) | 81.82 (0.62) | 80.51 (0.64) | 81.95 (0.28) |
| Average RUGS Case Mix Index | 0.84 (0.01) | 0.82 (0.01) | 0.84 (0.01) | 0.82 (0.00) |
| Average ADL | 16.59 (0.15) | 16.15 (0.33) | 16.39 (0.27) | 16.21 (0.14) |
| Percent high CPS | 17.93 (0.74) | 16.86 (1.35) | 16.99 (1.17) | 17.44 (0.69) |

*Notes*. BM=below median; A/A M= at/above median; RN=registered nurse; LPN=licensed practical nurse; CNA=certified nursing assistant; P4P=Medicaid pay for performance; RUGS=Resource Utilization Group; ADL=activities of daily living; CPS=cognitive performance scale
